# Letter to the Editor

**Published:** 1911-10

**Authors:** K. C. Russell

**Affiliations:** Washington, D. C.


### Letter to the Editor.

(PROHIBITION, THE CONSTITUTION, THE DECLARATION OF INDEPENDENCE, AND THE SUPREME COURT.—D.)

*Editor Texas Medical Journal:*

It is held by some that the State has no right to prohibit the liquor traffic because, forsooth, it would be an interference with the natural and inalienable right of each citizen. We take issue with that claim, and will cite the principles contained in the Declaration of Independence, which we believe will apply:

"We hold these truths to be self-evident; that all men are created equal; that they are endowed by their Creator with certain inalienable rights; *that among these are life, liberty, and the pursuit of happiness.* That to secure these rights governments are instituted among men, deriving their just powers from the consent of the governed, and whenever any form of government becomes destructive of these ends, it is the right of the people to alter or abolish it and to institute a government, laying its foundation on such principles and organizing its powers in such form as to them shall seem most likely to affect their safety and happiness."

We feel safe in concluding that inasmuch as the "life, liberty, and the pursuit of happiness" are to be fostered and protected by civil government, the State is acting wholly within her rightful province in prohibiting a business which in reality invades the natural or inalienable rights of the citizen. It can not be successfully denied that the liquor business is a menace to life, liberty, and the pursuit of happiness.

Many claim that the arguments of those who favor prohibition consist largely of sentiment. Not only do the facts refute such a claim, but decisions of the United States Supreme Court attest that the question is one of serious importance. In the case of Crowley vs. Christensen, 137 U. S., 86, the United States Supreme Court held:

"The statistics of every State show a greater amount of crime and misery attributable to the use of ardent spirits obtained at these liquor saloons than to any other source."

Again: "It is undoubtedly true that it is the right of every citizen of the United States to pursue any lawful trade, or business, under such restrictions as are imposed upon all persons of the same age, sex, and condition. * * * There is no inherent right in a citizen thus to sell intoxicating liquors by retail. It is not a privilege of a citizen of the State or of a citizen of the United States."

In the case of the State of Kansas vs. Zihold & Hahelin, 123 U. S. Rep., 623-662, the Supreme Court held:

"We can not shut out of view the fact within the knowledge of all that the public health, the public morals, and the public safety may be endangered by the general use of intoxicating drinks."

The United States Supreme Court has also held in License Cases, 5 Howard, 46 U. S., 573-632:

"If a loss of revenue should accrue to the United States (because of prohibition) from a diminished consumption of ardent spirits, she will be a gainer a thousand fold in the health, wealth, and happiness of the people."

Some hold tenaciously to the statement that prohibition does not prohibit. This statement is usually indulged in by those who are either in the liquor traffic or favor it. If this be true, why does the liquor element work so strenuously in opposition to prohibitory measures? But their claim may be swept to the winds by suggesting that we repeal all laws against murder, theft, assaults, and crimes of all character, because of the fact that these laws do not prohibit these crimes.

It was a grand day when the slavery of human beings was abolished in 1864. It will perhaps be as grand and important a day when the liquor traffic, which is making many more slaves than existed before the war, shall be eliminated by national and State government.

Very respectfully,

Washington, D. C. K. C. Russell.

